# Comparative Analysis of Bacterial Conjunctivitis in the Adult and Pediatric Inpatient vs. Outpatient Population

**DOI:** 10.3390/microorganisms13030473

**Published:** 2025-02-20

**Authors:** Adela Voinescu, Corina Musuroi, Monica Licker, Delia Muntean, Silvia-Ioana Musuroi, Luminita Mirela Baditoiu, Dorina Dugaesescu, Romanita Jumanca, Mihnea Munteanu, Andrei Cosnita

**Affiliations:** 1 Doctoral School, “Victor Babes” University of Medicine and Pharmacy, 300041 Timisoara, Romania; adela.voinescu@umft.ro (A.V.); silvia.musuroi@umft.ro (S.-I.M.); 2 Department of Microbiology, Multidisciplinary Research Center of Antimicrobial Resistance, “Victor Babes” University of Medicine and Pharmacy, 300041 Timisoara, Romania; licker.monica@umft.ro (M.L.); muntean.delia@umft.ro (D.M.); baditoiu.luminita@umft.ro (L.M.B.); dugaesescu.dorina@umft.ro (D.D.); 3 Clinical Laboratory, “Pius Brinzeu” Emergency Clinical County Hospital, 300723 Timisoara, Romania; 4 Department of Clinical Practical Skills, “Victor Babes” University of Medicine and Pharmacy, 300041 Timisoara, Romania; 5 Epidemiology Department, “Victor Babes” University of Medicine and Pharmacy, 300041 Timisoara, Romania; 6 Romanian and Foreign Languages Department, “Victor Babes” University of Medicine and Pharmacy, 300041 Timisoara, Romania; romanita.jumanca@umft.ro; 7 Department IX, Surgery and Ophthalmology, “Victor Babes” University of Medicine and Pharmacy, 300041 Timisoara, Romania; mihnea.munteanu@umft.ro (M.M.); cosnita.dan@umft.ro (A.C.)

**Keywords:** bacterial conjunctivitis, antimicrobial resistance, *Staphylococcus*, *Klebsiella*, *Pseudomonas*, *Acinetobacter*

## Abstract

The etiology and resistance pattern of bacterial conjunctivitis varies depending on the patient’s care setting and age. A retrospective, observational study was conducted in a tertiary care teaching hospital. A total of 126 patients—76 adults and 50 children—diagnosed with conjunctival infection during inpatient or ambulatory care were analyzed. In the samples of adult patients, isolates were represented by Gram-positive cocci (57.7%; *Staphylococcus* spp., *S. pneumoniae*) followed by *Enterobacterales* (17.97%; *P. mirabilis*, *E. coli*, *Klebsiella* spp.), and non-fermenters (7.69%; *Pseudomonas* spp., *A. baumannii*). Multidrug-resistant (52.17%) and extensively drug-resistant (21.73%) pathogens (predominantly Gram-negative bacilli) were identified in conjunctival swabs of hospitalized adult patients. The main isolates (55.77%) identified in children’s conjunctival swabs belonged to *S. aureus*, *H. influenzae*, and *S. pneumoniae*, followed by *Enterobacterales* (19.22%; *E. coli*, *P. mirabilis*, *M. morganii)* and fungi (3.48%). Methicillin-resistant *S. aureus* (35.71%) and extended-spectrum beta-lactamase-producing *K. pneumoniae* (8.7%) were identified in the pediatric subgroup of patients. In critically ill adult patients assisted in the intensive care or burn functional units, bacterial conjunctivitis followed the pattern of infections and antimicrobial resistance specific to these categories of patients. In the case of hospitalized children, conjunctivitis was an integral part of the age-related pathology.

## 1. Introduction

Adult conjunctivitis is an infection of the ocular surface that can be caused by bacteria, viruses, fungi, and parasites. Almost 80% of the total acute cases of conjunctivitis are of viral etiology, with adenovirus, herpes simplex virus, herpes zoster, and enterovirus [1] being more commonly involved.

Bacterial conjunctivitis has a lower frequency, being encountered more often in infants, school-age children, and the elderly, and the pathogens involved vary according to the age of the patient. In the case of adults, the most common bacterial conjunctival infections (CI) are caused by *Staphylococcus* spp., especially *S. aureus*, followed by *Streptococcus pneumoniae* and *Haemophilus influenzae* [2]. In the case of children, the disease is more often caused by *H. influenzae*, *S. pneumoniae*, and *Moraxella catarrhalis* [3]. Although fungal and parasitic infections are less common, they are more commonly described in immunocompromised people or after ocular trauma.

The epithelial lining of the conjunctiva is the main defense mechanism against infection, and any disruption of this barrier can lead to infection. Secondary defense mechanisms include the immune response via immunoglobulins, the presence of lysozymes in the tear film, conjunctival vascularization, and the rinsing action of tears [4].

There are data showing that newborns are more susceptible to conjunctivitis because of their lower lysozyme and IgA levels, as well as insufficient tear formation. Hospital-acquired conjunctivitis involving Gram-negative pathogens, such as *Klebsiella pneumoniae*, has been described in the neonatal intensive care units in patients exposed to invasive medical devices and with severe illnesses who are more likely to get healthcare-acquired infections [5].

Conjunctivitis may be caused by contamination from infected individuals, contact with pathogen-carrying hands, contamination at birth (e.g., gonococcal infection), indirectly by contact with contaminated surfaces, or may result from abnormal proliferation of commensal conjunctival flora. Along with these common causes, bacterial conjunctivitis can be caused by certain conditions such as dysfunctional tear syndrome, with alteration and disruption of the natural conjunctival epithelial barrier, abnormalities of the adjacent structures, as well as by the immunosuppressed state of the patient, i.e., trauma, severe burns, and hospitalization in intensive care units (ICUs) [6].

An alteration of eye protective mechanisms was observed in patients hospitalized in the ICU with low levels of consciousness, especially those with mechanical ventilation. In these patients, the loss of the blink reflex, with the eye open, and the lack of moisturization and antimicrobial protection provided by the tear film may lead to dry corneal epithelium and other superficial ocular disorders [7].

Conjunctivitis in patients with traumatic brain injury, especially those hospitalized in the ICU, is important because of the complex interaction with factors that contribute to maintaining trophicity and decreasing the risk of ocular surface infection. Traumatic brain injury can disrupt normal cranial vascular structures and, consequently, their tributary ocular functions. Complications such as carotid cavernous fistulas, a type of abnormal communication between the carotid artery and the cavernous sinus, can lead to conjunctival hyperemia, edema, and associated ocular symptoms. These vascular abnormalities lead to increased ocular venous pressure, manifesting in symptoms such as swollen conjunctivitis and infection [8].

As far as patients with severe burns are concerned, the increased survival rate has led to the identification of long-term complications related to eye injuries caused by the action of the traumatic factor itself or subsequent complications such as eye infections. Bouchard et al. [9] showed that almost 10% of patients admitted to the burn unit required an ophthalmological consultation, but only 50% of these consultations were requested on the day of admission. Underlying this observation is the fact that ocular complications are sometimes not evident at the time of admission, and the assessment of the patient is of particular interest in life-threatening issues.

Eye infections can be produced by mono- or polymicrobial biofilms, mostly associated with risk factors, including contact lenses or other ocular devices, surgery, trauma, age, and dry or previous eye disease. The ability of microbes to form biofilms also increases the rate of antimicrobial resistance (AMR) [10].

Data on eye infections in patients from our geographical region are limited, and most studies include infectious keratitis etiology and resistance patterns or mechanisms of eye infections associated with contact lenses [11,12,13,14].

Therefore, this study aims to fill this gap and to investigate and compare CI patterns in hospitalized versus ambulatory patients, in terms of etiology and AMR, in adult versus pediatric patients, with the aim of improving the clinical management of CI and enhancing the understanding of AMR dynamics in different patient populations.

## 2. Materials and Methods

A hospital-based, retrospective, observational study was conducted from January 2016 to October 2024 in the Clinical County Emergency Hospital “Pius Brînzeu”, Timisoara (SCJUT), Romania. This is a tertiary care teaching hospital (the largest university hospital in Western Romania) providing medical services for adult and pediatric patients; it has a capacity of 1174 beds and a specialized outpatient clinic. The hospital provides specialized medical care to patients with ophthalmology conditions only on an outpatient basis and does not have a ward with beds for this specialty.

During the period mentioned above, we conducted a comparative study of CI diagnosed in patients who were either referred to the Ophthalmology or Pediatric outpatient clinic of the hospital or were admitted to various wards of the hospital for other causes of illness.

The study was carried out on a total of 126 patients—76 adults and 50 children—diagnosed with CI during inpatient care (InC) or ambulatory care (AmC).

**Figure 1:**
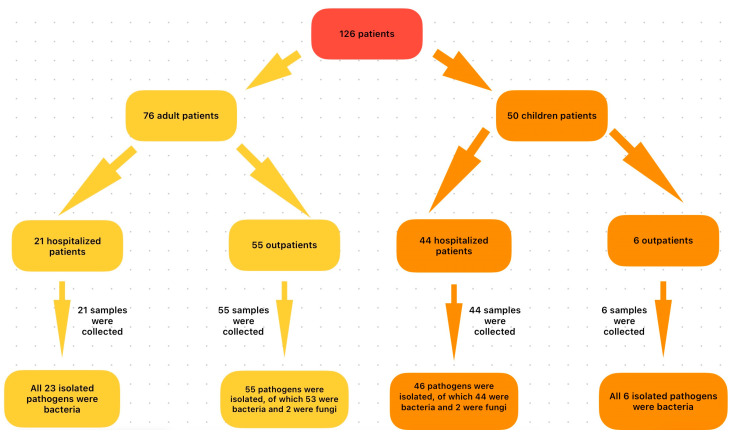
Study design.

In the group of adult patients (AD), 55 conjunctival samples from AD-AmC and 21 samples from AD-InC were analyzed. Out of these, 55 isolates (53 bacterial and 2 fungal) were identified in the AD-AmC group and 23 bacterial isolates were identified in the AD-InC group. On the other hand, in the pediatric (P) group, 6 samples collected from outpatients (P-AmC) and 44 samples from hospitalized children (P-InC) were analyzed. Out of these, 6 bacterial isolates were identified in the P-AmC group, and 46 isolates (44 bacterial and 2 fungal) were identified in the P-InC group (Figure 1).

We used the following inclusion criteria: positive bacterial cultures from conjunctival swabs of patients (irrespective of age) hospitalized or seen in the outpatient clinic during the study period. Duplicate samples with the same identified pathogen from the same patient were excluded.

### 2.1. Study Protocol

All the enrolled patients’ medical records were examined. Among the information gathered were age, gender, admission ward, length of hospitalization, comorbidities, other specimens collected, etiology of infections, antimicrobial susceptibility testing (AST), and antibiotics administered.

### 2.2. Microbiological Diagnosis

Samples were processed according to SCJUT Microbiology laboratory procedures. Conjunctival swabs were collected on flocked swabs with liquid Amies in a plastic screw cap tube (Copan Diagnostics, Brescia, Italy), transported to the laboratory, and inoculated on culture media (Thermo Fisher Scientific, Wesel, Germany). Identification was performed on VITEK^®^ 2 Compact (BioMerieux, Marcy l’Etoile, France) and matrix-assisted laser ionization/desorption on-flight mass spectrometry (MALDI Biotyper, Bruker, Brenen, Germany) systems. AST was performed either on VITEK^®^ 2 Compact, with determination of the minimum inhibitory concentration (MIC), or by the Kirby–Bauer disk diffusion method. Until 2022, interpretation of the AST was performed according to the Clinical and Laboratory Standards Institute (CLSI) [15]; however, since the beginning of 2023, it has been based on the European Committee on Antimicrobial Susceptibility Testing (EUCAST) breakpoints [16].

Clinically significant microorganisms were categorized according to acquired resistance phenotypes: Methicillin-resistant *S. aureus* (MRSA), Penicillinase (PASE)-producing organisms, Inducible Macrolide–Lincosamide–Streptogramin B resistance (MLSBi), Extended-spectrum beta-lactamases (ESBLs)-producing Gram-negative bacilli (GNBs), and Carbapenem-resistant organisms (CROs). Multidrug-resistant (MDR) bacteria were defined as having acquired resistance mechanisms to at least one antibiotic from three or more classes of active antibiotics for a particular species, and extensively drug-resistant bacteria (XDR) were defined as being resistant to at least one agent from all antimicrobial classes except one or two classes [17]. Antifungal susceptibility testing was not an objective in this study.

### 2.3. Statistical Analysis

Statistical analysis of data was conducted with EPI INFO version 7.2.50. Numerical variables were defined by mean and standard deviation (SD), and category variables were defined by value and percentage. Nominal variables were compared using the hi^2^ test (Fisher exact test) and their association was measured by calculating the Lambda Coefficient. The statistical significance threshold value was ≤0.05.

## 3. Results

### 3.1. Characteristics of the Study Group

The group of AD patients with CI consisted of 76 persons, of whom 46 (60.52%) were male (M) and 30 (39.48%) were female (F), aged between 19 and 87 years. The average age was 60 years, with 67.10% of patients being over 60 years of age. In this group, 55 people (72.36%) were AD-AmC, diagnosed with CI in the Outpatient Service, and 21 (27.64%) were inpatients (AD-InC) admitted in different wards in the hospital.

It was observed that the AD-AmC subgroup had the same M/F ratio (62.96%, 37.04%) and age distribution (mean age 65.92 years, 85.19% over 60 years) as the whole AD group. Compared with these, there was no statistically significant difference in M/F incidences in the AD-InC group (57.15%/42.84%) (*p* = 0.795), but the mean age was significantly lower (44.81 years) and 80.95% of the individuals were of working age (under 60 years).

The hospitalization wards of AD-Inc patients were Intensive Care Units (ICUs; 15.79%), Burns Functional Units (BFU; 6.58%), Obstetrics and gynecology (OG; 2.63%), and Neurology/Neurosurgery (NS; 2.63%).

AD-InC patients (21) were hospitalized for different disease conditions, as shown in Table 1, and CI was diagnosed during hospitalization. These patients’ disease diagnoses were of medium–high severity, revealed by the high number of hospitalization days (612 days), representing the total cumulative for all 21 AD-InC patients, with a mean number of 29.14 hospitalization days/patient. Furthermore, 11 (52.38%) required continuous ventilatory support (=/<96 h). Only one patient with diabetes mellitus and another one with hypertension were reported; the rest of the patients had no history of a chronic disease.

The total number of days of antibiotic treatment for the AD-InC patients was 347 days, with an average of 16.95 days/patient, and the average number of antibiotics administered was 3.24/patient.

Patients in the AD-AmC group (55) required medical assistance only for CI, and no other infections were reported at the time of the outpatient clinic visit.

The pediatric patient group consisted of 50 children—24 M (48%) and 26 F (52%). Among them, 70% were children up to 1 year of age (32% newborns and 38% infants (1 month–1 year)), 20% were children 1–3 years old, and 10% were older than 3 years. Most children diagnosed with CI were P-InC (88%), admitted to the neonatology (32%) and pediatric ward (56%), according to the age at admission. Only 12% were P-AmC, cared for in the outpatient system.

In the group of pediatric patients, the P-AmC subgroup was small, consisting of six children, all up to 3 years of age. At the time of presentation, they showed no other clinical signs of disease, and no further paraclinical investigations were performed.

The group of hospitalized children was larger (44). Their hospitalization diagnosis is presented in Table 2.

In the P-InC subgroup, the total number of hospitalization days was 441, with a mean of 10 days/patient, and the mean number of antibiotics administered was 1.57/patient. In 38.63% of the cases, local treatment in the form of topical antibiotic eye drops was also applied.

### 3.2. Etiology of Conjunctival Infections

In the AD patient group, a total of 76 samples were studied, with 78 bacterial/fungal isolates identified. The predominant strains were Gram-positive cocci (GPC) (61.54%) followed by Gram-negative bacilli (GNB) (32.05%), with a low presence of Gram-positive bacilli (GPB) (3.85%) and fungi (2.56%). This distribution was also observed in the AD-AmC subgroup samples.

On the contrary, the situation was different for the AD-InC group, where the predominant isolates were GNB (56.52%), followed distantly by GPC (34.78%).

In the pediatric group of patients, 52 conjunctival specimens were studied, with 52 bacterial/fungal isolates identified. The most numerous were GPC (48.07%), followed by GNB (28.85%), GN coccobacilli (GNCB) (19.23%), and rare fungal strains (3.85%). The frequency patterns were the same in the two subgroups (P-AmC and P-InC), with GPC dominating.

### 3.3. Distribution of Bacterial/Fungal Species

The bacterial/fungal species distribution in adult conjunctival samples is presented in Table 3.

In the samples from AD patients, bacterial isolates were richly represented by *Staphylococcus* spp. (*S. aureus*, CNS) and *S. pneumoniae*, with a total of 57.7%, followed by *Enterobacterales* (*P. mirabilis*, *E. coli*, *Klebsiella* spp.; 17.97%) and non-fermenters (*Pseudomonas* spp., *A. baumannii*; 7.69%). The diversity of species was high, especially on account of GNB, but their presence was low in terms of number of strains. The presence of fungi was reported to be low in frequency.

In the AD-AmC subgroup, *Staphylococcus* spp. (predominantly *S. aureus*) and *S. pneumoniae* accounted for 72.71% of the entire bacterial population. GNB represented only 20%, having no significance in this subgroup. Two *C. albicans* isolates were also identified (Table 3).

The isolates identified in the conjunctival swabs of AD-InC samples are shown in Table 3. In this subgroup, a balanced distribution of species was noted, with non-fermenters (*P. aeruginosa*, *A. baumannii*) making up 21.72%, *Enterobacterales* (*Klebsiella* spp., *E. coli*, *P. mirabilis*) making up 34.78%, and GPC (*S. aureus*, CNS, *Enterococcus* spp., *Streptococcus* spp.) making up 34.79% of the bacteria.

AD-InC were distinguished from outpatients by the high number of diverse microbiologically positive specimens associated with positive conjunctival samples: 76.19% of them presented bacterial infections at other locations, with an average number of 4.37 positive samples (4.13 pathogens/patient). In these cases, the hospitalization wards were ICU and BFU, where 91.66% and 100% of AD patients with CI were treated, respectively. Furthermore, 61.90% of patients with multiple positive samples had the same bacterial species as identified in conjunctival secretion in at least one other sample (wound secretions, bronchial aspirate, blood culture). Also, the same AMR pattern was noticed in some of the isolates (Table 4).

The Lambda coefficient indicated a significant association between germs identified in conjunctival secretions and germs from bronchial aspirates (=0.563, *p* = 0.006), but especially with germs from wound secretions (=0.737, *p* ≤ 0.001).

A total of 52 isolates were identified in children’s conjunctival exudates, as presented in Table 5.

The main bacterial isolates identified in the conjunctival swabs of pediatric patients belonged to *S. aureus*, *H. influenzae*, and *S. pneumoniae* (55.77%), followed by *Enterobacterales (E. coli*, *P. mirabilis*, *M. morganii*, *C. koseri*—19.22%) and fungi (*Candida albicans*—3.48%). *Moraxella osloensis* and *Pasteurella pneumotropica* were sporadic isolates. However, the presence of the non-fermenters *A. baumannii* (9.61%) and *Moraxella osloensis* (1.92%) was also noted.

The number of children seen as outpatients was small. Therefore, the bacterial isolates identified in the conjunctival samples of hospitalized children followed the distribution pattern presented for the entire group of pediatric patients. *H. influenzae* and *S. pneumoniae* had the highest frequencies (54.36%), but *A. baumannii* (10.87%) and *Klebsiella* spp. (8.7%) were also present (Table 5).

### 3.4. Antibiotic Resistance and Acquired Resistance Phenotypes

The results of AMR testing are presented in Table 6A (for GN pathogens) and Table 6B (for GP pathogens). In AD-InC patients, the species with aggressive behavior both in antibiotic resistance range and frequency were *A. baumannii* and *P. aeruginosa* (Table 6A). Among the GP bacteria, *S. aureus* isolates exhibited widespread resistance across numerous antibiotic classes, but the phenomenon did not represent the majority in the total number of strains identified. The *K. pneumoniae* isolates showed a moderate pattern of resistance to cephalosporins without posing treatment problems. The GNB (*Proteus* spp., *E. coli)* and GPC (CNS and *Enterococcus* spp.) showed outstanding resistance phenotypes.

In AD-AmC samples, isolates were susceptible to antibiotics. AMR of the GN group to sulfonamide testing (SXT) was noticeable. Testing of GP isolates showed broad resistance to penicillin (PEN) (*S. aureus*, CNS, *S. pneumoniae*) and Erythromycin (E) (*S. aureus*, CNS) and a medium frequency of resistance to Cefoxitin (FOX) (*S. aureus*, CNS MR isolates).

In the P-InC patients, aggressive AMR was noticed in *K. pneumoniae* through resistance to cephalosporins and SXT. Also, in the group of GP species, a similar behavior was observed for *S. pneumoniae*, with resistance to multiple drugs (PEN, E, Clindamycin (DA), Tetracycline (TE), SXT), as well as *S. aureus* isolates (resistance to PEN, FOX, E, Ciprofloxacin (CIP)), with medium frequencies of resistance in the studied group.

In the group of outpatient children, *E. coli* showed resistance to cephalosporins and fluoroquinolones, and *S. pneumoniae* isolates had high resistance levels, similar to P-InC isolates, while *S. aureus* was sensitive to FOX (non-MRSA strains).

### 3.5. Acquired Resistance Phenotypes

The comparative analysis of the resistance phenotype distribution by patient category is presented in Table 7.

In the case of adult patients, the isolates identified in the samples of hospitalized patients presented multiple resistance mechanisms, whose association led to high percentages of MDR (52.17%) and XDR (21.73%) strains due to *A. baumannii*, *P. aeruginosa*, *S. aureus* isolates. The comparative study of AD-InC/total AD emphasized that AMR was significantly higher in hospitalized patients for all the AMR phenotypes: MDR (*p* = 0.018), XDR (*p* = 0.002), ESBL (*p* < 0.001), and CRO (*p* = 0.002). The AD-InC/AD-AmC comparison indicated the same phenomenon: a significantly increased presence of ESBL (*p* = 0.015) and CRO (*p* = 0.039) in the AD-InC group. No significant differences in MRSA phenotypes were recorded (*p* = 0.431).

In the case of pediatric patients, MDR strains were identified in almost one-third of the P-InC, especially because of the resistance of GP strains. These are associated with multiple AMR phenotypes (FQ, PASE, MR, MLSB), and *S. aureus*, *S. pneumoniae*, and ESBL-producing *K. pneumoniae* strains were noted in this regard. The comparative study P-InC/P-AmC indicated that there were no significant differences in AMR phenotypes between the two groups (MDR *p* = 0.659, ESBL *p* = 1, MRSA *p* = 0.324).

## 4. Discussion

Adult infectious conjunctivitis rarely causes permanent loss of vision or structural damage, but the economic impact of the disease in terms of lost activity time, cost of diagnostic tests, and treatment is considerable [6], both for the patient with simple conjunctivitis as well as in the case of ocular complications associated with other medical conditions.

The present study performed a comparative analysis of conjunctivitis in adult and pediatric inpatients and outpatients with positive bacteriological results of conjunctival swabs.

In the case of outpatient adults, conjunctivitis was more frequent in male patients, particularly in those over 60 years of age (85.19%). For AD patients, no general anamnestic data (medical history, current health status, and risk factors) or specific data (eye diseases, infectious or non-infectious) were recorded; hence, this distribution can be explained by the higher risk behavior of men (social behavior, disease) compared to women [18,19].

In comparison, hospitalized adults had a relatively equal gender distribution and a significantly lower mean age at admission (*p* < 0.001), with 80% of people aged up to 60 years.

These characteristics were determined by the fact that most of these patients were hospitalized because of a major acute accidental event (trauma, burns), characteristic of the active, mature population.

The etiology of conjunctival infections in AD-InC patients was as expected, in most cases determined by GPC (72.72%) represented by *Staphylococcus* spp. and *S. pneumoniae*. Regarding hospitalized adults, more than half of the strains (56.52%) were GNB, with a significant presence of *K. pneumoniae*, *P. aeruginosa*, and *A. baumannii*, with high AMR potential.

Comparatively, in another Romanian study [11], CNS, *S. aureus*, and *P. aeruginosa* were the most frequent causes of eye infections (keratitis) in adult ambulatory patients. High levels of resistance to clindamycin and gentamycin, as well as minimal quinolone resistance, were recorded in GP bacteria, while amoxicillin–clavulanic acid and ampicillin were not effective against the GNB, but third-generation cephalosporins, carbapenems, and quinolones were efficient.

Regarding pediatric patients, this study showed that the highest incidence of conjunctival infections was recorded in children up to 1 month of age, in a context of illness for which medical care was provided through hospitalization (Table 2). The most affected were newborns, who presented risk factors specific to this age, such as perinatal hypoxia, prematurity, maternal–fetal contamination, and an immature immune system (as in other studies [20]). Children up to 1 month of age were affected at a lower frequency; however, in their case, the conjunctival infection was included in a broader acute infection context of ear, nose, throat, or pulmonary infection.

The number of children who were consulted as outpatients was small compared to that of hospitalized children, and 2/3 of them were older than 1 month.

The incidence of isolates in the total pediatric group, as well as the subgroups (hospitalized/non-hospitalized), followed the infection pattern known for this age, with mainly *S. aureus*, *S. pneumoniae*, and *H. influenzae* being identified. GNB frequencies were comparable between subgroups (P-AmC/P-Inc), with values varying at around 30%. In this context, the *K. pneumoniae* isolates identified in children under 1 month of age had a special significance. In three of the four cases of infection, the children’s pathology was of high severity. The first case was of a child with prematurity grade II and intraventricular hemorrhage, the second case was of a child with SARS-CoV-2 infection, and the third was a newborn with intraamniotic infection. All strains were ESBLs.

There are numerous data in the literature citing *Staphylococcus* spp. [21,22,23], but also *K. pneumoniae* [5,22,23] or other GNB, such as *P. aeruginosa*, *Serratia marcescens*, and *Escherichia coli* [24,25] as etiological agents in community or hospital-acquired conjunctivitis.

Regarding the AMR and the presence of the acquired resistance phenotypes, this study highlights that the infections in hospitalized patients (adults or children) were caused by strains with high resistance behavior.

In our study, most hospitalized patients required assistance to maintain vital functions, including assisted ventilation, central venous catheterization, and urinary catheterization, and most of them underwent major surgical interventions starting with the first day of hospitalization. Two-thirds of the conjunctival secretions were collected and found to be positive in the first 3 days of hospitalization, thus underlining the major immunocompromised status of these patients for whom contamination was rapid, with strains with increased AMR.

Moreover, approximately 69% of conjunctival isolates from AD-InC patients were also identified in other samples collected from the same patients, showing that the ocular infection was an extension of other infectious sites, mostly from the skin, in the case of burn patients, and from tracheobronchial aspirates, in the case of trauma patients hospitalized in the ICU.

The XDR isolates were *A. baumanii*, *K. pneumoniae*, and *P. aeruginosa*, all in samples from ICU patients. One *S. aureus* isolate sensitive only to glycopeptides (teicoplanin, vancomycin) and linezolid was also identified in an ICU patient with septic skin necrosis and subsequent septic shock. In this regard, Manente et al. showed that in the conjunctival samples studied, the most frequent GP isolates were CNS, followed by *S. aureus*. *Pseudomonas* spp. and *E. coli* were the most frequent GN species. In general, linezolid, teicoplanin, tigecycline, and vancomycin were the most effective antimicrobials. However, an increase in resistance to azithromycin, clarithromycin, and erythromycin was noted among CNS, and clindamycin and erythromycin among *S. aureus* [26].

A recent study conducted by our team on a group of BFU patients showed that in this ward, *P. aeruginosa* and *S. aureus* were prevalent species in wound secretions, while *A. baumannii*, *Pseudomonas* spp., and *S. maltophilia* prevailed in bronchial aspirates [27].

Data from the literature show that in ICU patients, the eye becomes colonized with bacteria as the length of hospital stay increases [28]. Up to 77% of mechanically ventilated patients are colonized with at least one abnormal bacterial species within 7–42 days, and 40% of those with prolonged ventilation and sedation are colonized with multiple bacteria. The most common isolates are *P. aeruginosa*, *Acinetobacter* spp., and *S. epidermidis* [29]. Respiratory secretions are the major source of ocular surface infection, with aerosols from tracheal suction and direct contact from suction catheters both implicated. *Pseudomonas* infection rates may be reduced if endotracheal suction is performed from the side (rather than the head) of the patient and with the eyes covered [30].

In the present study, isolates from AD-InC patient samples showed a high AMR profile, with 52.17% MDR and 21.73% XDR/CRO, with the main species with this behavior being *A. baumannii* (CRO/XDR), *P. aeruginosa* (CRO/XDR), and *K. pneumoniae* (ESBL/CRO/XDR). Also, 50% of *S. aureus* isolates were MRSA.

In the case of hospitalized children, MDR strains reached an incidence of 30.43%, of which approximately two-thirds were *Staphylococcus* spp. *S. aureus* conjunctivitis was important both numerically and by resistance behavior. Of these, 78.57% were PASE-producing strains and 35.71% were MRSA. Conjunctivitis cases caused by GNB were relatively low (almost 30% of the total), but their importance was given by the fact that one-third of the strains were ESBL, all of them *K. pneumoniae*.

MDR pathogens from conjunctival swabs of long-term hospitalized patients are often biofilm-forming. Prolonged hospitalization increases exposure to MDR bacteria skilled at creating biofilms on mucosal surfaces or medical devices [31,32]. Biofilms enhance pathogen survival and resistance to eradication, especially in case of hospital-acquired infections or the use of contact lenses. MDR pathogens often utilize quorum sensing and surface adhesins to form biofilms, which act as barriers against immune responses and treatments [12,13]. The ability of many pathogens to form ocular biofilms is well known in the case of *Staphylococcus* spp. [21,33,34], *K. pneumoniae* [5], or other GNBs [25].

In the case of *S. aureus* causing microbial keratitis and conjunctivitis, a high prevalence of isolates carrying *mecA* genes encoding methicillin resistance was found by other authors [35], emphasizing the role of their identification in treatment decisions. Furthermore, identification of both the *ermB* and *mecA* genes is crucial in the case of MDR *S. aureus*, offering insights for better treatment strategies [36].

In this regard, we conducted molecular testing of isolates, and in the case of two *S. aureus* strains from the AD-Inc subgroup, we identified one strain carrying *mecA* and the other carrying *ermB*/*gyr83* (conferring macrolide/fluoroquinolone resistance). In both cases, AST was confirmed by the presence of the resistance genes.

In terms of therapeutic options, antibiotic selection should be guided by the specific pathogens and their AMR patterns. In outpatients, local treatment with antibiotic-based eye drops (with aminoglycosides and fluoroquinolones) is usually preferred. However, we noticed a relatively high percentage of resistance to aminoglycosides in AD-Amc patients (33.34%) and to fluoroquinolones in P-Amc patients (33.33%). On the contrary, in hospitalized patients, the underlying infectious pathology dictates the therapeutic options. When dealing with MDR/XDR bacteria, the choices are limited to parenteral back-up antibiotics (such as carbapenems or vancomycin).

In our study, 26 (59%) of 44 hospitalized children received systemic antibiotic therapy (e.g., ampicillin, cefuroxime, ceftriaxone, gentamicin, amikacin, meropenem, vancomycin) for underlying conditions, with nine of these also receiving adjunctive local treatment. A total of 16 children (36.7% of those hospitalized) were treated locally with eye drops. The most frequently used local therapies included Nettacin (Netilmicin), Betabioptal (Betamethasone, Chloramphenicol), and Tobradex (Dexamethasone, Tobramycin).

Tailoring treatment to the local AMR profile is critical for optimal outcomes. There are many views on this subject in the literature [37], suggesting that while chloramphenicol or fluoroquinolone drops are effective for acute conjunctivitis in children, they are often unnecessary, as most cases resolve without antibiotics. Parents are advised to clean the eyes and monitor symptoms, consulting a doctor only if symptoms persist or worsen, reducing antibiotic overuse and resistance [38].

Conversely, other data support topical antibiotics for bacterial conjunctivitis to shorten illness duration, alleviate discomfort, and prevent reinfection, though frequent application can lead to poor compliance in children [3,39]. Also, factors such as availability, allergies, AMR, and cost influence the choice of treatment [3]. According to other authors, 1.5% azithromycin eye drops are effective in children, offering quicker cures, fewer doses, good safety, and tolerability, even for newborns [40]. It shortens treatment duration and improves compliance, especially in pediatric cases. On the other hand, in severe infections caused by MR *Staphylococcus*, vancomycin remains the treatment of choice if MRSA is associated with multiple resistance mechanisms, while standard treatments for *P. aeruginosa* and *H. influenzae* remain effective due to their low resistance rates in community-acquired infections [41].

To prevent ocular complications in ICU patients, some authors proposed a protocol based on eyelid closure and individual risk factors [42,43]. It includes regular saline eye cleaning, frequent use of lubricants like Hypromellose, and protective measures tailored to eyelid position. Fully closed eyelids require minimal care, while partial or full corneal exposure needs intensive lubrication and protective taping. Combined with regular monitoring and supportive practices, these measures significantly reduce the risk of dry eyes, conjunctivitis, and corneal ulcers, ensuring better visual outcomes.

This is the first study on CIs in our geographical area and has tried to contribute to the improvement of the clinical management of bacterial conjunctivitis and enhance our understanding of AMR dynamics in different patient populations. However, we concede that the low number of samples analyzed from a single hospital, as well as the very low number of molecular tests performed, could represent some study limitations. Also, the classification of the XDR phenotype was limited to the number of antibiotics tested. Future research directions include the characterization of the molecular patterns of all pathogens involved, as well as their biofilm-forming potential.

## 5. Conclusions

By understanding the local AMR patterns, clinicians can make evidence-based decisions on initial empiric treatments for CI based on the patient’s care setting. In our study, conjunctivitis in outpatients was mainly caused by Gram-positive pathogens, with no increased rates of resistance and local treatment with antibiotic-based eye drops being preferred. On the contrary, in hospitalized patients, especially in critically ill adult patients in the ICU or BFU, bacterial conjunctivitis followed the pattern of infections and AMR specific for these categories of patients. Their etiology was determined predominantly by Gram-negative pathogens with high resistance behavior, which complicated the patient’s evolution, and parenteral backup antibiotics were used. In the case of hospitalized children, conjunctivitis was an integral part of the age-related pathology; however, we noticed the worrying situation of *S. aureus* isolates with increased rates of MRSA phenotypes and the presence of ESBL-producing GNB, which required systemic antibiotic therapy.

## Figures and Tables

**Table 1:** Disease conditions of hospitalized adult patients.

Pathology	Nr. (%from21)	MVP
Craniocerebral trauma with massive cerebral contusions +/− facial bone fractures	8 (38.10%)	7 (33.34%)
Hemorrhagic stroke	2 (9.52%)	2 (9.52%)
Burn wounds on the face, GR IIA-IIB-III	5 (23.81%)	-
Sepsis, septic shock (sepsis infections with skin/urinary starting point)	2 (9.52%)	1 (4.76%)
Thoracic crush injury with acute respiratory failure and resuscitated cardiac arrest	1 (4.76%)	1 (4.76%)
Tumor pathology of the orbit	1 (4.76%)	-
Pregnancy with emergency cesarean section	2 (9.53%)	-
Total	21 (100%)	11 (52.38%)

Legend: MVP = mechanically ventilated patients.

**Table 2:** Disease conditions and age of hospitalized children.

Pathology	Total	Newborns	Infants	1–3 Years Old	Older than 3 Years
Neonatal conjunctivitis and dacryocystitis *	13 (29.54%)	13 (29.54%)	-	-	-
Acute intrapartum hypoxia	3 (6.82%)	3 (6.82%)	-	-	-
Infectious diseases **	14 (31.82%)	1 (2.27%)	8 (18.18%)	-	5 (11.36%)
Sepsis	3 (6.82%)	-	-	2 (4.55%)	1 (2.27%)
SARS-CoV-2	4 (9.09%)	1 (2.27%)	3 (6.82%)	-	-
Cystic fibrosis	4 (9.09%)	1 (2.27%)	1 (2.27%)	2 (4.55%)	-
Others ***	3 (6.82%)	-	-	-	3 (6.82%)
Total	44	19 (43.17%)	12 (27.27%)	4 (9.09%)	9 (20.45%)

Legend: * Associated with neonatal jaundice, ** Infection disease: Pneumonia, Acute Bronchiolitis. Otitis media, Tonsillitis, Laryngitis, Others ***: Malnutrition, Measles, Portal vein thrombosis.

**Table 3:** Bacterial/fungal isolates distribution in conjunctival samples of adult patients.

	SA	CNS	PM	EC	P spp.	K spp.	SP	AB	CA	Oth *
AD strains % (78)	38.46%	15.38%	7.7%	6.41%	5.13%	3.86%	3.86%	2.56%	2.56%	14.08%
AD-AmC strains % (55)	50.9%	16.36%	7.27%	3.64%	1.82%	0%	5.45%	0%	3.64%	10.92%
AD-InC strains % (23)	8.7%	13.04%	8.7%	13.04%	13.04%	13.04%	0%	8.7%	0%	21.74%

Legend: SA = *S. aureus*, CNS = Coagulase-negative staphylococci, PM = *P. mirabilis*, EC = *E. coli*, P spp. = *Pseudomonas* spp., K spp. = *Klebsiella* spp., SP = *S. pneumoniae*, AB = *A. baumannii*, CA = *C. albicans*, Oth * = Others: *Morganella morganii*, *Bacillus cereus*, *Enterococcus* spp., *Citrobacter koseri*, *Enterobacter aerogenes*, *Serratia marcescens*, *Corynebacterium striatum*, *Streptococcus* spp.

**Table 4:** Identical bacterial species identified in conjunctival secretions and other samples with antimicrobial resistance patterns.

AD-Inc	Ward	Same Strains from Conjunctival Swabs and Other Samples	Resistance Phenotypes in Conjunctival Swabs	Other Samples	Resistance Phenotypes in Other Samples
AD1	ICU	AB	XDR, ESBL, CRO	Bronchial aspirate	XDR, ESBL, CRO
AD2	ICU	AB	MDR, CRO, ESBL	Bronchial aspirate	MDR, CRO ESBL
AD3	ICU	PA	XDR, CRO	Bronchial aspirate	XDR, CRO
AD4	ICU	SA	MDR, MRSA	Nasal exudate	MDR, MRSA
AD5	ICU	PA	NRF	Bronchial aspirate	MDR
AD6	ICU	K spp.	NRF	Wound secretion	MDR
AD7	ICU	PM	MDR	Wound secretion	MDR
AD8	ICU	K spp.	XDR, ESBL, CRO	Bronchial aspirate	XDR, ESBL, CRO
AD9	BFU	Strep. spp.	NRF	Wound secretion	NRF
AD10	BFU	EC	NRF	Wound secretion	NRF
AD11	BFU	E. spp.	MDR	Wound secretion	MDR
AD12	BFU	E. spp., BC	NRF	Bronchial aspirate	NRF
AD13	ICU	K spp.	MDR	Blood culture	MDR, ESBL

Legend: AD 1–13—adult hospitalized patients, ICU—intensive care unit, BFU—burns functional unit, AB—*A. baumanii*, PA—*P. aeruginosa*, SA = *S. aureus*, K spp.—*Klebsiella* spp., Strep. spp.—*Streptococcus* spp., EC—*E. coli*, PM—*P. mirabillis*, CB—*Citrobacter brakii*, BC—*Bacillus cereus*, E. spp.—*Enterococcus* spp., NRF—no acquired resistance phenotype, MDR = multidrug-resistant, XDR = extensively drug-resistant, ESBL = extended-spectrum beta-lactamases, CRO = Carbapenem-resistant organisms.

**Table 5:** Distribution of bacterial/fungal strains in conjunctival samples from pediatric patients.

	SA	HI	SP	AB	K spp.	CNS	EC	CA	MO	Oth *
P strains % (52)	30.77%	15.38%	9.62%	9.62%	7.69%	5.77%	5.77%	3.84%	1.92%	9.62%
P-AmC Strains % (6)	33.34%	16.67%	16.6%	0%	0%	0%	16.67%	0%	0%	16.67%
P-InC Strains % (46)	30.43%	15.23%	8.7%	10.8%	8.7%	6.52%	4.35%	4.35%	2.17%	8.68%

Legend: SA = *S. aureus*, HI = *H. influenzae*, SP = *S. pneumoniae*, AB = *A. baumannii*, K spp. = *Klebsiella* spp., CNS = Coagulase-negative staphylococci, EC = *E. coli*, CA = *C. albicans*, MO = *Moraxella osloensis*, Oth * = Others: *Pasteurella pneumotropica*, *Proteus* spp., *M. morganii*, *Citrobacter koseri*, *Enterococcus* spp.

**Table 6:** (A) Resistance of GN strains to antibiotics (%R); (B) Resistance of GP strains to antibiotics (%R).

(A)															
R ≥ 60%	R 35–59%	R < 35%													
AD-InC	AMP	AMC	TZP	CXM	CAZ	CRO	FEP	IMI	MEM	GM	AK	CIP	LVX	SXT	COL
*A. baumannii*			100		100		100	100	100	100	100	100		100	0
*E. coli*		100	33.3	0	0	0	0	0	0	0	0	0	0	50	0
*Klebsiella* spp.		100	66.7	66.7	33.3	66.7	33.3	33.3	33.3	0	0	33.3	33.3	33.3	0
*Proteus* spp.			0		100	0	0	0	0	100	0	50	0	0	
*P. aeruginosa*		66.7	66.7	100	66.7		66.7	66.7	66.7	66.7	67	66.7			0
AD-AmC															
*E. coli*		50		0	0	0	0	0	0	0		0		50	
*Proteus* spp.		0		0	0	0	0			0		0		100	
*P. aeruginosa*			0		0			0		0		0			
*M. morganii*		0		0	0	0	0			50		0	0	100	
P-InC															
*H. influenzae*	60	33.34	0	33.4	0	0	0	25	0	0	0	0	0	40	
*Klebsiella* spp.		75	25	100	100	100	100	0	0	0	100	25	25	66.7	
*A. baumannii*			0		50		50	0	0	0	0	60		0	0
P-AmC															
*H. influenzae*		0				0						0	0	0	
*E. coli*	100	100	0		100	100	100		0	100	0	100	100		
(B)															
AD-InC	PEN	AMP	FOX	CRO	CIP	LVX	GM	E	DA	TE	SXT	LZD	TEC	VA	
SA	100		50		50		50	50	50		50	0	0		
CNS	100		0		0	0	33.3	66.7	33.4		50	0	0		
*Enterococcus* spp.		50				0	0					0		0	
AD-AmC															
SA	92		34.6		32.0	0	38.0	73.7	32		20.8	0	0	0	
CNS	87.5		33.3		33.4		37.5	71.4	11.1		28.6	0	0		
SP	66.7			0				33.4	0		0	0	0	0	
P-InC															
SA	78.6		38.5		55.6		0	38.5	23.1		18.2	0	0	0	
CNS	100		50		100		33.4	33.4			50	0	0	0	
SP	100			0		0		75	50	66.7	75	0			
P-AmC															
SA	S0		0		0		0	50	50			0	0		
SP	100			0		0		100	100	0	100	0			

Legend: SA = *S. aureus*, CNS = Coagulase negative staphylococci, SP = *S. pneumoniae*, AMP = Ampicillin, AMC = Amoxicillin/Clavulanic acid, TZP = Piperacillin/Tazobactam, CXM = Cefuroxime, CAZ = Ceftazidime, CRO = Ceftriaxone, FEP = Cefepime, IMI = Imipenem, MEM = Meropenem, GM = Gentamicin, AK = Amikacin, CIP = Ciprofloxacin, LVX = Levofloxacin, SXT = Trimethoprim/Sulfamethoxazole (Cotrimoxazole), COL = Colistin, PEN = Penicillin, FOX = Cefoxitin, E = Erythromycin, DA = Clindamycin, TE = Tetracycline, LZD = Linezolid, TEC = Teicoplanin, VA = Vancomycin.

**Table 7:** Distribution of acquired resistance phenotypes in adult and pediatric patient groups.

					GNB		*Staphylococcus* spp.		
	MDR	XDR	R-AG	R-FQ	ESBL	CRO	PASE	MR	MLSBi
AD	32.05%	6.41%	37.09%	23.52%	7.69%	6.41%	32.05%	12.82%	12.82%
AD-AmC	23.64%	0%	33.34%	15.56%	0%	0%	38.18%	16.36%	14.54%
AD-InC	52.17%	21.73%	45%	39.13%	26.08%	21.73%	17.39%	4.34%	8.69%
P	28.85%	0%	2.7%	31.81%	7.69%	0%	36.53%	15.38%	15.38%
P-AmC	16.67%	0%	0%	33.33%	0%	0%	16.67%	16.67%	33.34%
P-InC	30.43%	0%	3.03%	31.57%	8.7%	0%	39.13%	15.22%	13.04%

Legend: MDR = multidrug-resistant, XDR = extensively drug-resistant, ESBL = extended-spectrum beta-lactamases, CRO = Carbapenem-resistant organisms, PASE = Penicillinase, MR = Methicillin-resistant, MLSBi = Inducible Macrolide–Lincosamide–Streptogramin B resistance, GNB = Gram-negative bacilli, R-AG = aminoglycosides resistance, R-FQ = fluoroquinolones resistance.

## Data Availability

The original contributions presented in this study are included in the article. Further inquiries can be directed to the corresponding author.

## Abbreviations

The following abbreviations are used in this manuscript:

CI	Conjunctival infection
ICU	Intensive care unit
AMR	Antimicrobial resistance
InC	Inpatient
AmC	Ambulatory care
AD	Adult patients
P	Pediatric patients
P-AmC	Outpatient service (pediatric)
P-InC	Hospitalized children
AST	Antimicrobial susceptibility testing
MIC	Minimum inhibitory concentration
CLSI	Clinical and Laboratory Standards Institute
EUCAST	European Committee on Antimicrobial Susceptibility Testing
MRSA	Methicillin-resistant Staphylococcus aureus
PASE	Penicillinase-producing organisms
MLSBi	Inducible Macrolide–Lincosamide–Streptogramin B resistance
ESBLs	Extended-spectrum beta-lactamases-producing Gram-negative bacilli
CROs	Carbapenem-resistant organisms
MDR	Multidrug-resistant
XDR	Extensively drug-resistant bacteria
SD	Standard Deviation
M	Male
F	Female
BFU	Burns Functional Unit
NS	Neurosurgery
GPC	Gram-positive cocci
GNB	Gram-negative bacilli
GPB	Gram-positive bacilli
GNCB	Gram-negative coccobacilli
CNS	Coagulase-negative staphylococci
